# PTPN2 regulates the activation of KRAS and plays a critical role in proliferation and survival of KRAS-driven cancer cells

**DOI:** 10.1074/jbc.RA119.011060

**Published:** 2021-01-13

**Authors:** Zhangsen Huang, Mingzhu Liu, Donghe Li, Yun Tan, Ruihong Zhang, Zhizhou Xia, Peihong Wang, Bo Jiao, Ping Liu, Ruibao Ren

**Affiliations:** Shanghai Institute of Hematology, State Key Laboratory for Medical Genomics, National Research Center for Translational Medicine at Shanghai, Collaborative Innovation Center of Hematology, Ruijin Hospital affiliated to Shanghai Jiao Tong University School of Medicine, Shanghai, China

**Keywords:** PTPN2, KRAS, ERK, tyrosine phosphatase, plasma membrane, cell proliferation, cell signaling, GTPase Kras (KRAS), extracellular-signal-regulated kinase (ERK), tyrosine-protein phosphatase (tyrosine phosphatase)

## Abstract

*RAS* genes are the most commonly mutated in human cancers and play critical roles in tumor initiation, progression, and drug resistance. Identification of targets that block RAS signaling is pivotal to develop therapies for *RAS*-related cancer. As RAS translocation to the plasma membrane (PM) is essential for its effective signal transduction, we devised a high-content screening assay to search for genes regulating KRAS membrane association. We found that the tyrosine phosphatase PTPN2 regulates the plasma membrane localization of KRAS. Knockdown of PTPN2 reduced the proliferation and promoted apoptosis in KRAS-dependent cancer cells, but not in KRAS-independent cells. Mechanistically, PTPN2 negatively regulates tyrosine phosphorylation of KRAS, which, in turn, affects the activation KRAS and its downstream signaling. Consistently, analysis of the TCGA database demonstrates that high expression of PTPN2 is significantly associated with poor prognosis of patients with KRAS-mutant pancreatic adenocarcinoma. These results indicate that PTPN2 is a key regulator of KRAS and may serve as a new target for therapy of *KRAS*-driven cancer.

RAS proteins are small GTPases that regulate diverse cellular processes, including proliferation, differentiation, migration, apoptosis, and senescence (1). Mammalian cells mainly express three *RAS* genes that encode four highly homologous proteins: HRAS, NRAS, KRAS4A, and KRAS4B. KRAS4A and KRAS4B result from an alternative splicing at the C terminus of the *KRAS* gene (2). Because KRAS4B is the predominant splice variant of *KRAS*, it is referred to as KRAS hereafter.

*RAS* genes are the most frequently mutated oncogene in cancer, nearly 20-30% of human malignancies carry RAS gene mutations. Among the *RAS* gene family, *KRAS* is the most commonly mutated, which occurs in 71% of pancreatic, 29% of colorectal, and 18.6% of lung carcinomas (3). It has been shown that mutated *KRAS* not only plays pivotal roles in cancer initiation (4, 5, 6), but also contribute to several hallmarks of human cancer (7, 8). Moreover, inhibition of activated KRAS could delay tumor progression both *in vitro* and *in vivo* (9, 10, 11). These observations prompted many groups to target either mutant KRAS directly or downstream effectors.

Thus far, directly targeting oncogenic *KRAS* only succeeded in one certain form, KRAS^G12C^ (12, 13), which comprises only 12% of *KRAS* mutations in all human cancers, so it is still needed to develop new target molecules for other oncogenic KRAS. Inhibiting protein–protein interactions and KRAS localization are novel approaches to target mutant KRAS and block oncogenic KRAS signaling (14, 15, 16), although the efficacy in clinical is still unknown. Targeting the KRAS effector signaling pathways could also prove efficacious in treating tumors with *KRAS* mutations, as the inhibitors have entered clinical trials demonstrating promising clinical activity in *KRAS* mutant tumor (17, 18). However, toxicities associated with their sustained inhibition, variable responses rates, and acquired adaptive resistance due to the activation of other kinases are limiting the efficacy and the clinical progression of these compounds as monotherapy treatment (19, 20, 21). Hence it remains an urgent need to identify new target strategies to block oncogenic KRAS signaling. KRAS interacts with downstream effectors only when it associates with the plasma membrane (PM) (14, 22, 23, 24), so inhibition of KRAS localization is a valid therapeutic approach to block signal transmission by oncogenic KRAS.

KRAS is synthesized as cytosolic proteins and gains affinity for the PM through post-translational modification of its carboxyl terminal C*AAX* motif (CVIM sequence) by farnesyltransferases (FTase) or geranylgeranyltransferase (GGTase), followed by cleavage of the VIM residues by RAS converting enzyme 1 (RCE1) and methyl esterification of the farnesylated cysteine residue by isoprenylcysteine carboxyl methyltransferase (ICMT) (22). Given the critical role of prenylation for KRAS membrane association and neoplastic transformation, and farnesylation of KRAS by FTase is the first step in the KRAS post-translational modification, FTase is the ideal target for KRAS-driven cancers. However, FTase inhibitors (FTIs) failed to show high efficacy as expected in clinic (25). The main reason is that KRAS protein can undergo alternative prenylation by GGTase in the presence of FTIs (26). Inhibitors targeting FTase and GGTase in combination have been proved too toxic to be clinically useful (27).

Despite the clinical failure of FTIs, inhibition of KRAS–PM interactions remains an attractive therapeutic approach to abrogate the KRAS oncogenic activity (14, 28, 29, 30, 31). Here we devised a high-content screening assay and carried out an siRNA screening to identify key molecules required for oncogenic KRAS plasma membrane association. We show that, for the first time, protein-tyrosine phosphatase non-receptor type 2 (PTPN2) regulates the KRAS plasma membrane association and plays an important role in KRAS-dependent cancer cell proliferation and survival. Mechanistic studies demonstrate that PTPN2 negatively regulates tyrosine phosphorylation of KRAS, which, in turn, affects the activation KRAS and its downstream signaling. Our data suggest that PTPN2 could serve as a potential therapeutic target for KRAS-driven cancer.

## Results

### Identification of PTPN2 as a regulator for KRAS plasma membrane association by an siRNA screening

**Figure 1:**
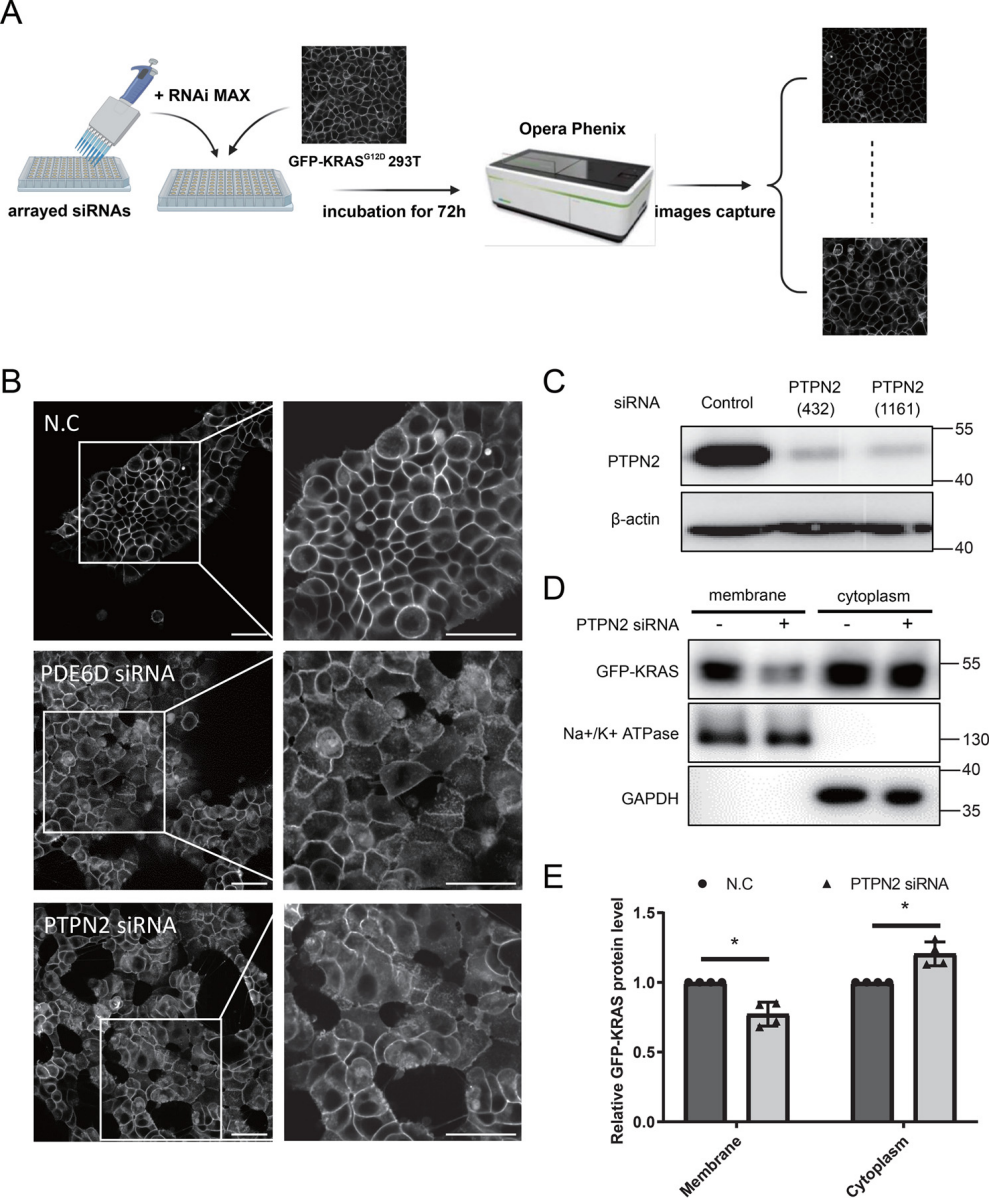
**PTPN2 regulates KRAS membrane association.***A,* schematic representation of the screening. Arrayed siRNAs were transfected into GFP-KRAS^G12D^ 293T using RNAiMAX. After the 72-h incubation, images were acquired for 17 fields with a ×40 water immersion objective using the GFP-confocal mode in an Opera Phenix High-Content Screening System. *B,* PTPN2 siRNA induced GFP-KRAS^G12D^ mislocalization. Representative images (GFP channel only) of GFP-KRAS^G12D^ HEK 293T cells transfected with PTPN2 smartpool ON-TARGETplus siRNAs. *Scale bar*: 50 µm. *C,* Western blot analysis of the knockdown efficiency of PTPN2 in GFP-KRAS^G12D^ HEK 293T after transfection with the indicated siRNAs. The immunoblot data are representative of at least three independent experiments. *D,* GFP-KRAS^G12D^ HEK 293T cells were treated with pooled PTPN2 siRNAs or NC siRNA for 72 h. To detect exogenous GFP-fused KRAS proteins, membrane proteins, and cytoplasmic proteins were isolated, followed by Western blot analysis with an anti-GFP antibody. (Na^+^-K^+^)-ATPase was used as the input of membrane proteins control, and GAPDH was used as the input of cytoplasmic proteins control. *E,* quantitation of data in *C* are shown. Signal intensity was quantified for GFP-KRAS^G12D^ in the membrane fraction and cytoplasmic fraction (*n* = 4 independent biological experiments depicted as normalized intensity for siPTPN2 over the NC control; mean ± S.D.; two-tailed *t* test. *, *p* < 0.05).

To identify genes required for KRAS membrane trafficking, we developed an image-based screening assay that monitors the degree of KRAS^G12D^ membrane association (Fig. 1*A*). To this end, we established a GFP-fused KRAS^G12D^ (GFP-KRAS^G12D^) human embryonic kidney (HEK) 293T cell line stably expressing GFP-KRAS^G12D^. We found that the majority of GFP-KRAS^G12D^ proteins are localized to the PM (Fig. 1*B*). We then screened a 147-siRNA sublibrary enriched with targets that regulate protein translocation, using an Opera Phenix High-Content Screening System (the siRNA-target genes and sequence was listed in Table S1). The PDE6D siRNA was used as a positive control. After cells were treated with arrayed siRNAs for 72 h in 96-well–plates, one siRNA, PTPN2 siRNA, significantly changed the ratio of PM-bound GFP-KRAS^G12D^ over cytosolic GFP-KRAS^G12D^ (Fig. 1*B*).

We used additional PTPN2 siRNA, as well as KRAS siRNA, from GenePharma to confirm the screening result. Two different PTPN2 siRNAs (PTPN2 siRNA-432 and PTPN2 siRNA-1161) could effectively decrease the level of PTPN2 in GFP-KRAS^G12D^ HEK 293T cells (Fig. 1*C*). We then checked the level of GFP-KRAS^G12D^ on the PM using a membrane protein extraction kit, and found that silencing PTPN2 reduced the translocation of KRAS^G12D^ to the PM by approximately 30% compared with the negative control (NC) (Fig. 1, *D* and *E*). Meanwhile, the level of KRAS^G12D^ in the cytoplasm was slightly increased (Fig. 1, *D* and *E*). These data suggest that PTPN2 plays an important role in regulating the plasma membrane association of KRAS^G12D^.

### PTPN2 is required for KRAS-dependent tumor cell growth

**Figure 2:**
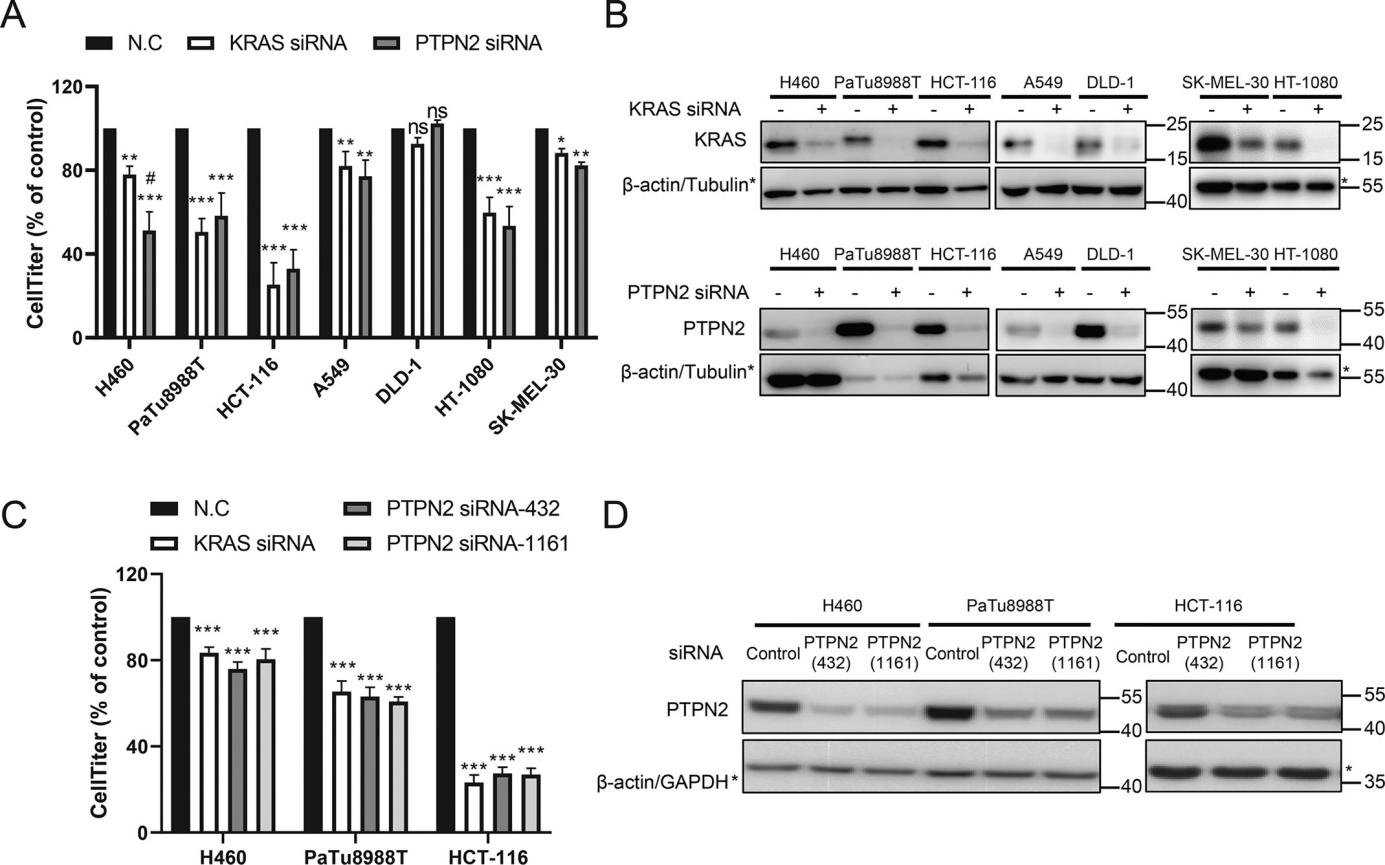
**Effect of PTPN2 knockdown on the viability of KRAS-dependent cells.***A,* H460, PaTu8988T, HCT-116, A549, DLD-1, HT-1080, and SK-MEL-30 cells were transfected with control siRNA and siRNA targeting KRAS or PTPN2, analyzed for proliferation by Cell Titer Glo 72 h later. Values plotted are mean ± S.D.; *n* = 3, one-way ANOVA followed by Tukey's test. *, *p* < 0.05 *versus* NC; **, *p* < 0.01 *versus* NC; ***, *p* < 0.001 *versus* NC; and #, *p* < 0.05 *versus* KRAS siRNA. *B,* H460, PaTu8988T, HCT-116, A549, DLD-1, HT-1080, and SK-MEL-30 cells were transfected with the indicated siRNAs, harvested, and lysates prepared on days 3 after transfection, and immunoblotted as indicated for KRAS, PTPN2, and β-actin/Tubulin. The immunoblot data are representative of at least three independent experiments. *C,* H460, PaTu8988T, and HCT-116 cells were transfected with control siRNA, KRAS siRNA or two different PTPN2 siRNAs, and analyzed for proliferation by Cell Titer Glo 72 h later. Values plotted are mean ± S.D.; *n* = 3; one-way ANOVA followed by Tukey's test. **, *p* < 0.01 *versus* NC; ***, *p* < 0.001 *versus* NC. *D,* H460, PaTu8988T, and HCT-116 cells were transfected with the indicated siRNAs, harvested, and lysates were prepared on days 3 after transfection, and immunoblotted as indicated for KRAS, PTPN2, and β-actin/GAPDH. The immunoblot data are representative of at least three independent experiments.

We next investigated the effect of PTPN2 on cancer cell growth, using pooled siRNAs to knockdown PTPN2 in a panel of five mutant KRAS-harboring human cancer cell lines (H460, PaTu8988T, HCT-116, A549, and DLD-1) and two KRAS wildtype human cancer cell lines (HT-1080 and SK-MEL-30). Pooled siRNAs to knockdown KRAS were used as a positive control. Cells were treated with siRNAs for 72 h, followed by the CellTiter-Glo® luminescent cell viability assay. As shown in Fig. 2*A*, silencing PTPN2 with pooled siRNAs markedly attenuated the proliferation of H460 (lung), PaTu8988T (pancreatic), HCT-116 (colon), and HT-1080 (fibrosarcoma) cells, and slight but significantly growth inhibition of A549 (lung) and SK-MEL-30 (skin). However, it is not the case in DLD-1 (colon) cells. Interestingly, the growth inhibitory effect of knocking down PTPN2 was consistent with that of knocking down KRAS (Fig. 2*A*), suggesting that PTPN2 plays a critical role in KRAS oncogenic signaling. Accordingly, DLD-1, which is KRAS-independent, was less sensitive to the knockdown of either PTPN2 or KRAS (Fig. 2*A*). Both two pooled siRNAs effectively decreased the expression of PTPN2 and KRAS in all seven cell lines (Fig. 2*B*).

We further confirmed the effect of PTPN2 on the three mutant KRAS-dependent cell lines by using two different siRNAs, respectively. Both siRNAs were found to reduce the expression of PTPN2 and suppress the proliferation of the cell lines (Fig. 2, *C* and *D*).

### PTPN2 is required for KRAS-dependent tumor cell survival

**Figure 3:**
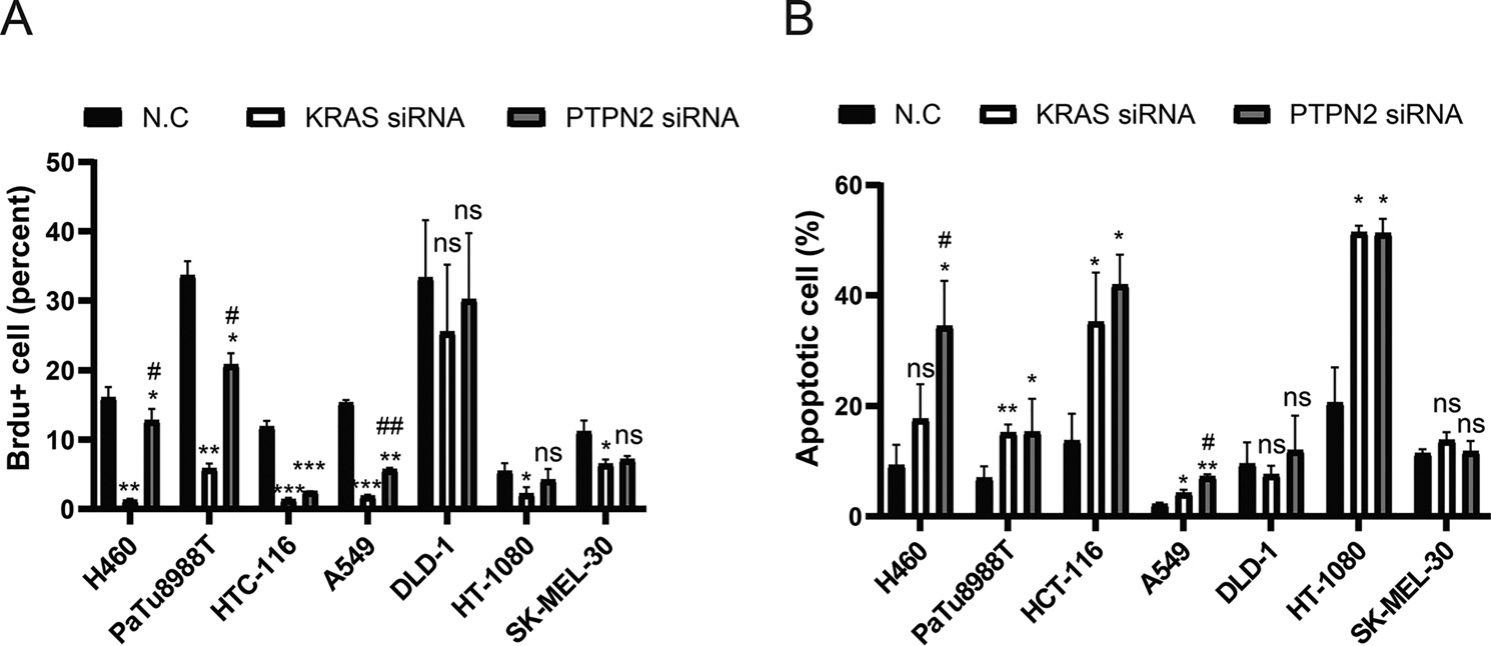
**Effect of PTPN2 knockdown on the proliferation and survival of KRAS-dependent cells.** H460, PaTu8988T, HCT-116, A549, DLD-1, HT-1080, and SK-MEL-30 were transfected with PTPN2 or KRAS siRNA and then the BrdU incorporation (*A*) and apoptosis (*B*) were measured by flow cytometry analyses. Data represent the mean ± S.D. of three independent experiments; one-way ANOVA followed by Tukey's test. *, *p* < 0.05 *versus* NC; **, *p* < 0.01 *versus* NC; #, *p* < 0.05 *versus* KRAS siRNA; ##, *p* < 0.01 *versus* KRAS siRNA. *ns*, non-specific.

For more accurate detection of cell proliferation, we examined the effect of PTPN2 and KRAS knocking down on cell proliferation by a BrdU assay. Consistent with the results of CellTiter-Glo® luminescent cell viability assay, the fraction of BrdU-positive cells was significantly decreased in all these cell lines described above except for DLD-1 (Fig. 3*A*). It is notable that the effect of PTPN2 knockdown is less than that of KRAS knockdown in H460, PaTu8988T, and A549, suggesting that PTPN2 has limited activity on the proliferation signaling of KRAS in these cells.

We also examined the effect of PTPN2 on cell survival. All these tumor cell lines described above were treated with PTPN2, KRAS, or control siRNAs for 72 h, followed by staining with Annexin V-APC and propidium iodide. Flow cytometric analysis showed that the percentage of apoptosis in these KRAS-dependent tumor cell lines was significantly higher than that of controls (Fig. 3*B*). The effect of PTPN2 knockdown is no less than that of KRAS knockdown in KRAS-dependent tumor cell lines, suggesting that PTPN2 is required for the cell survival signaling of KRAS.

### PTPN2 regulates the activation of oncogenic KRAS and its downstream signaling

**Figure 4:**
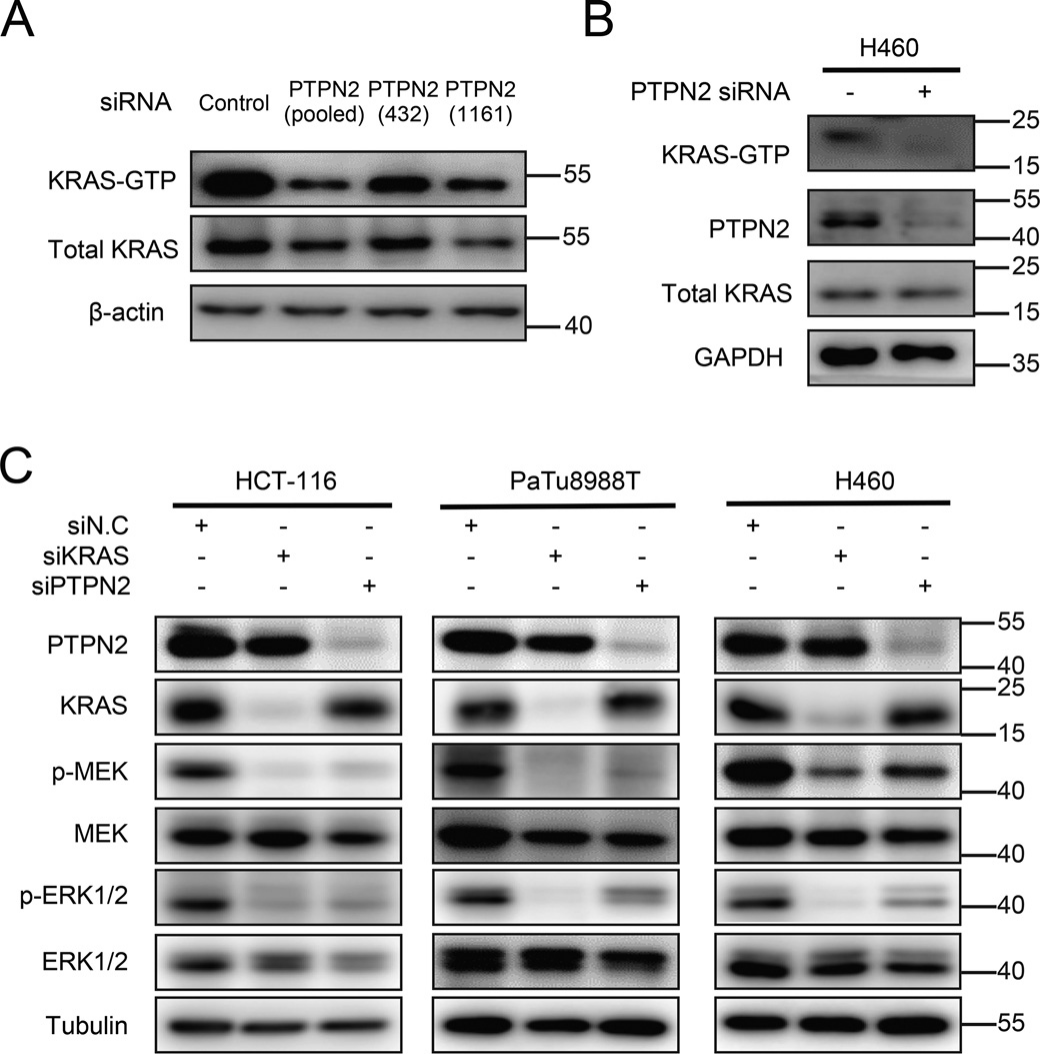
**Effect of PTPN2 knockdown on the KRAS activation, signaling, and tyrosine phosphorylation.***A,* GFP-KRAS^G12D^ 293T cells were transfected with PTPN2 siRNA or NC and cultured for 72 h. Cellular lysates were prepared and the levels of RAS–GTP were determined. Pulldown of RAS-GTP was performed with RAF-RBD-agarose beads. Precipitates were immunoblotted using an anti-KRAS antibody (labeled *KRAS-GTP*). The input samples were immunoblotted with the identical antibodies. *B,* H460 cells were transfected with PTPN2 siRNA or NC and cultured for 72 h. Cellular lysates were prepared and the levels of RAS–GTP were determined. Pulldown of RAS-GTP was performed with RAF-RBD-agarose beads. Precipitates were immunoblotted using an anti-KRAS antibody (labeled *KRAS-GTP*). The input samples were immunoblotted with the identical antibodies. The immunoblot data are representative of two independent experiments. *C,* HCT-116, PaTu8988T, and H460 cells were transfected with PTPN2 siRNA, KRAS siRNA, or NC and cultured for 72 h. Cells were lysed and immunoblotted with the indicated antibodies. The immunoblot data are representative of three independent experiments.

To gain insights into the mechanism by which PTPN2 regulates the proliferation and survival of KRAS-dependent tumor cells, we first checked whether PTPN2 affects the KRAS activation, using an RAS-GTP pulldown assay. As shown in Fig. 4*A*, the level of GTP-KRAS^G12D^ was significantly decreased in GFP-KRAS^G12D^ expressing 293T cells treated with PTPN2 siRNA, either pooled or two different single siRNAs, compared with that in GFP-KRAS^G12D^ expressing 293T cells treated with scrambled siRNA. Similarly, the level of GTP-bound KRAS in H460 cells was also decreased by treating with pooled PTPN2 siRNAs (Fig. 4*B*).

We next tested whether PTPN2 affects the activation of KRAS downstream signaling pathway. As shown in Fig. 4*C*, both KRAS and PTPN2 knockdown could dramatically decrease the phosphorylation levels of MEK and ERK in KRAS-dependent tumor cell lines HCT-116, PaTu8988T, and H460. Collectively, these data demonstrate that PTPN2 is required for the full activation of oncogenic KRAS and its downstream signaling pathway.

**Figure 5:**
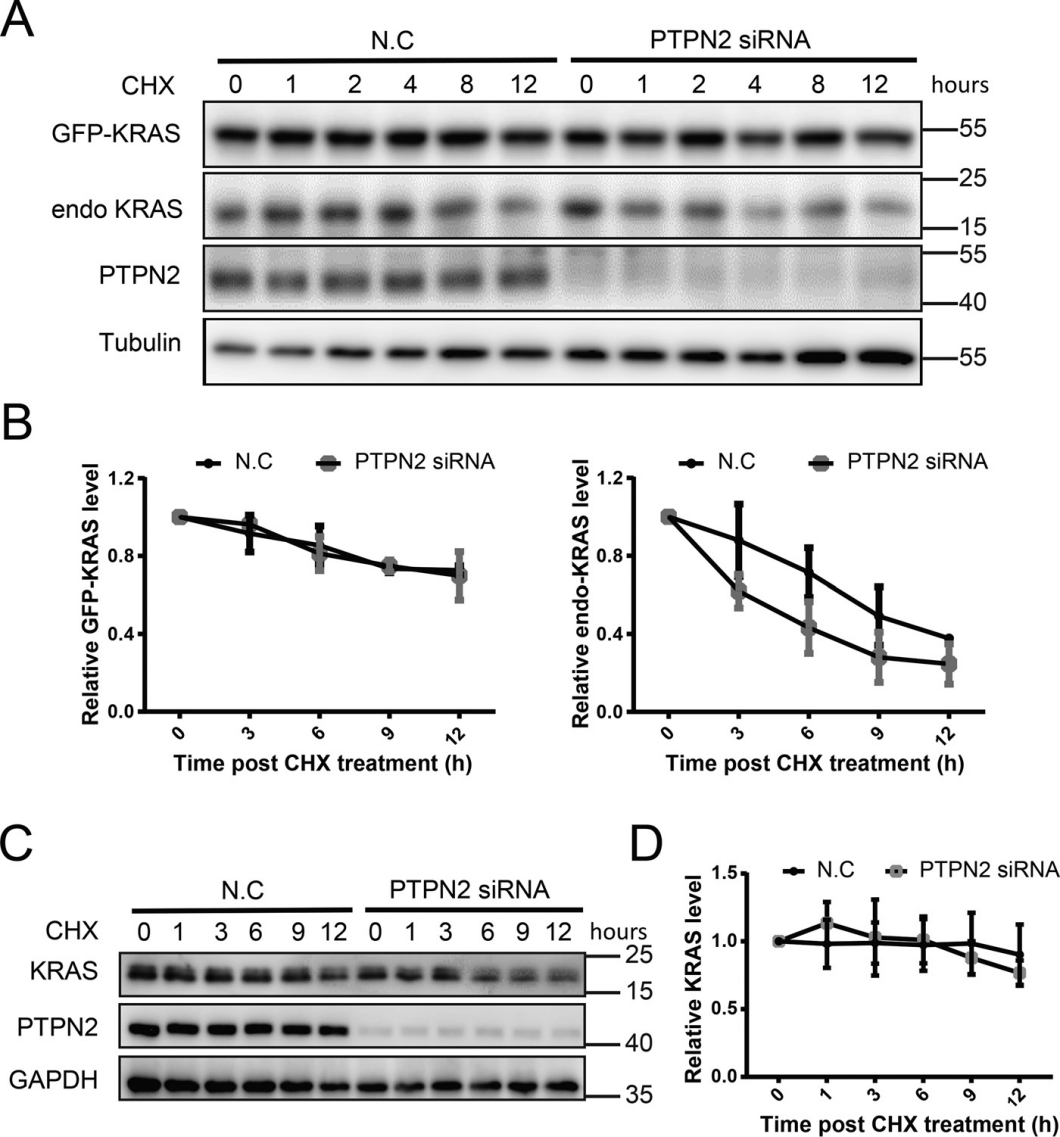
**PTPN2 regulates the endogenous KRAS protein stability.***A,* GFP-KRAS^G12D^ HEK 293T cells were transfected with the indicated siRNA. Forty-eight h post-transfection, cells were treated with CHX (50 µg/ml), and cell lysates were harvested at the indicated time points and analyzed by immunoblotting with the antibodies indicated. The immunoblot data are representative of two independent experiments. *B,* graphical representation of the quantification of exogenous (GFP-KRAS^G12D^) and endogenous KRAS protein levels shown in *A* to determine protein half-life. Relative KRAS levels were determined by densitometric scanning of the representative immunoblot. The band intensity at time 0 was set as 1 (arbitrary units). *C,* H460 cells were transfected with the indicated siRNA. Forty-eight h post-transfection, cells were treated with CHX (50 µg/ml), and cell lysates were harvested at the indicated time points and analyzed by immunoblotting with the antibodies indicated. The immunoblot data are representative of two independent experiments. *D,* graphical representation of the quantification of KRAS protein levels shown in *C* to determine protein half-life. Relative KRAS levels were determined by densitometric scanning of the representative immunoblot. The band intensity at time 0 was set as 1 (arbitrary units).

We also examined the protein stability of GFP-KRAS^G12D^ in HEK 293T cells in the presence of protein synthesis inhibitor cycloheximide (CHX, 50 μg/ml). As shown in Fig. 5*A*, the endogenous KRAS protein was markedly susceptible to PTPN2 knockdown, whereas only a marginal change was noted for the exogenous GFP-KRAS^G12D^ protein. The different effects of PTPN2 on the stability between endogenous and exogenous KRAS may be partly explained by the exogenous KRAS is the mutant form, whereas the endogenous KRAS is the wildtype form. We also examined the effect of PTPN2 knockdown on half-life of the endogenous KRAS protein in the KRAS mutant cell line. We transfected cancer cells carrying KRAS-Q61K (H460) mutant with either control or PTPN2 siRNA. As shown in Fig. 5, *C* and *D*, mutant KRAS half-life changed marginally in PTPN2 knockdown cells. Taken together, these data demonstrate that PTPN2 is not involved in maintaining mutant KRAS protein stability.

### PTPN2 regulates the level of tyrosine phosphorylation of KRAS

**Figure 6:**
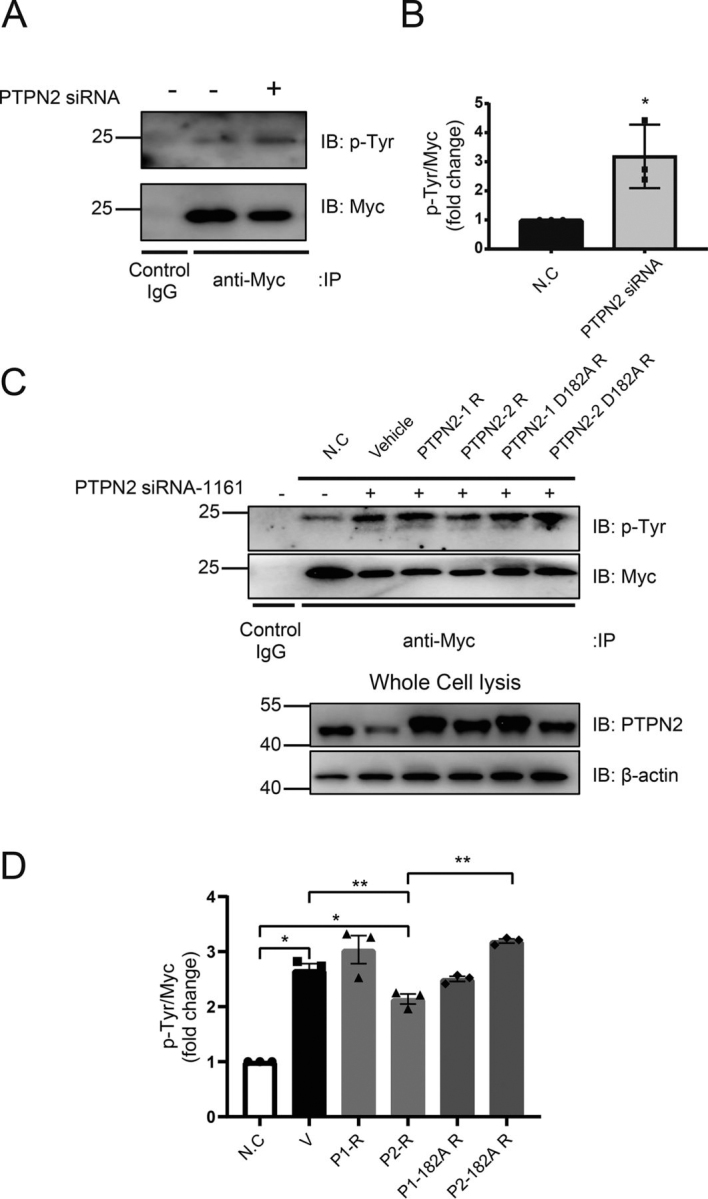
**Effect of PTPN2 knockdown on the KRAS tyrosine phosphorylation.***A,* HEK 293T cells were transfected with the indicated plasmid and siRNA. Cells were lysed, immunoprecipitated, and immunoblotted with the indicated antibodies. The immunoblot data are representative of three independent experiments. *B,* quantitation of data in *A*. Assessment of tyrosine phosphorylation of KRAS in samples from three repeat experiments by Western blot (*n* = 3; mean ± S.D.; two-tailed *t* test. *, *p* < 0.05). *C,* HEK 293T cells were transfected with the indicated plasmid and siRNA. Cells were lysed, immunoprecipitated (*IP*), and immunoblotted (*IB*) with the indicated antibodies. The immunoblot data are representative of three independent experiments. *D,* quantitation of data in *C*. Assessment of tyrosine phosphorylation of KRAS in samples from 3 repeat experiments by Western blot (*n* = 3; mean ± S.D.; one-way ANOVA followed by Tukey's test. *, *p* < 0.05; **, *p* < 0.01). P1-R, PTPN2-1 R; P2-R, PTPN2-2 R; P1-182A R, PTPN2-1 D182A R; P2-182A R, PTPN2-2 D182A R.

It has been shown that tyrosine phosphorylation of KRAS reduces its signal transduction activity, whereas tyrosine-phosphorylated KRAS can be dephosphorylated by SHP2 (encoded by PTPN11) (32). PTPN2 is a member of the PTP family and has been shown to dephosphorylate several tyrosine kinases. We tested whether PTPN2 regulates KRAS signaling through dephosphorylating KRAS. Myc-KRAS^G12D^ HEK 293T cell lysates were immunoprecipitated with anti-Myc antibody followed by anti-pTyr immunoblot. We found the level of phosphorylated KRAS in Myc-KRAS^G12D^ HEK 293T cells was significantly increased after knockdown of PTPN2 (Fig. 6, *A* and *B*). These results indicate PTPN2 is also a tyrosine phosphatase for KRAS. To confirm that the observed effect on KRAS phosphorylation was attributable specifically to PTPN2 knockdown, we generated two siRNA-resistant wildtype PTPN2 isoforms (PTPN2-1-R and PTPN2-2-R) and a phosphatase-inactive mutant (PTPN2-D182A) (Asp-182 changed to Ala) construct for rescue experiments (Fig. 6*C*). Transfection of PTPN2-1-R and PTPN2-2-R constructs into PTPN2 siRNA (1161)-treated cells markedly increased PTPN2 expression (Fig. 6*C*). More importantly, overexpression of PTPN2-2-Res but not PTPN2-2-D182A-Res, nor PTPN2-1-Res, decreased the level of tyrosine phosphorylation of KRAS (Fig. 6, *C* and *D*). This result indicates that PTPN2 regulates tyrosine phosphorylation of KRAS through its tyrosine phosphatase activity and that PTPN2-2-Res is the primary isoform to carry out this function.

### High level PTPN2 expression is associated with poor prognosis of pancreatic adenocarcinoma

**Figure 7:**
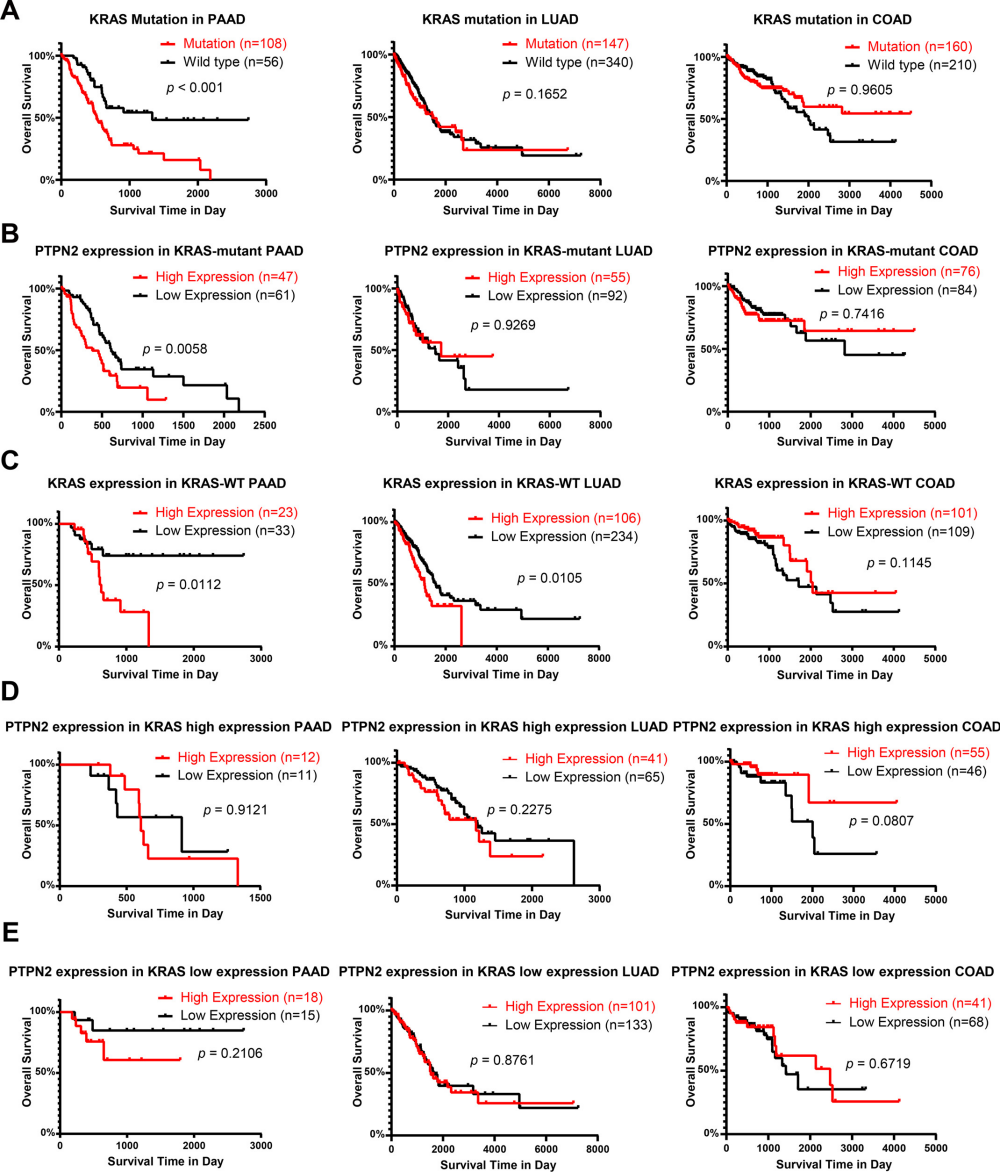
**High level expression of PTPN2 is associated with poor prognosis of KRAS-mutant PAAD patients.***A,* Kaplan-Meier plots of overall survival of patients with PAAD, LUAD, or COAD stratified by KRAS mutation. *B,* Kaplan-Meier plots of overall survival of patients with PAAD, LUAD, or COAD stratified by PTPN2 expression in KRAS-mutant cancers. *C,* Kaplan-Meier plots of overall survival of patients with PAAD, LUAD, or COAD stratified by KRAS expression in KRAS-WT cancers. *D,* Kaplan-Meier plots of overall survival of patients with PAAD, LUAD, or COAD stratified by PTPN2 expression in KRAS high expression cancers. *E,* Kaplan-Meier plots of overall survival of patients with PAAD, LUAD, or COAD stratified by PTPN2 expression in KRAS low expression cancers. Data were all obtained from the TCGA database.

Our data show that PTPN2 plays an important role in the maintenance of KRAS-dependent tumor cells. To determine the impact of PTPN2 expression in KRAS-related cancers, we analyzed clinical databases. We separated KRAS mutation, KRAS expression, PTPN2 expression, and clinical information of KRAS-related cancers from TCGA cohorts (Table S2). We first performed overall survival analysis to compare the survival of patients harboring mutant or wildtype KRAS in pancreatic adenocarcinoma (PAAD), lung adenocarcinoma (LUAD), and colorectal adenocarcinoma (COAD). As shown in Fig. 7*A*, KRAS mutation is associated with poor prognosis of PAAD, but not that of LUAD or COAD. We then analyzed the impact of PTPN2 on survival of patients containing KRAS mutations. As shown in Fig. 7*B*, high expression of PTPN2 is significantly associated with poor prognosis in KRAS-mutant PAAD patients (*p* = 0.0058), but not in patients with LUAD or COAD. In KRAS-WT patients, the high expression of KRAS mRNA is significantly associated with poor prognosis in PAAD (*p* = 0.0112) and LUAD (*p* = 0.0105) but not in COAD (*p* = 0.1145) (Fig. 7*C*). However, PTPN2 expression levels are not significantly associated with survival in KRAS high expression patients or KRAS low expression patients with PAAD (*p* = 0.9121 and *p* = 0.2106, respectively) (Fig. 7, *D* and *E*). These clinical data support our conclusion that PTPN2 plays an important role in KRAS-mutant–dependent tumors.

## Discussion

It has been known that 20-30% human cancers, including a high percentage of pancreatic, lung, and colorectal cancers, are driven by mutations in KRAS. Genetically engineered mouse models have shown that *Kras* mutation could faithfully recapitulate the progression of the human cancer, and a mutated form of *Kras* is sufficient to initiate pancreatic and lung cancer (33, 34). Furthermore, silencing *Kras* with siRNA in these genetically engineered mouse models significantly prolonged the survival of mice (9, 10, 11). Although scientists have made great progresses in the last 3 decades toward understanding the KRAS signaling pathways, drug development in blocking KRAS function has been ineffective. Based on the fact that KRAS regulates signaling pathways for cell growth only when associated with the PM (14, 22, 23, 24), targeting KRAS membrane translocation has become an alternative approach. In this study, we screened a custom-designed siRNA library using an image based high-content screening. We found that PTPN2 is required for the effective PM localization of KRAS, and PTPN2 is required for KRAS–dependent cell survival and, to a lesser extent, proliferation. Mechanistically, PTPN2-negative regulated KRAS tyrosyl phosphorylation. In addition, analysis of the data from TCGA, we found that high PTPN2 expression is associated with poor prognosis of KRAS-mutant PAAD patients.

*PTPN2*, also known as T cell protein-tyrosine phosphatase (TCPTP), is a non-receptor phosphatase that is ubiquitously expressed (35), and plays critical roles in T cell-mediated immunity and inflammation (36, 37, 38). The role of PTPN2 in tumors has just begun to be investigated in recent years. It has been reported that *PTPN2* is frequently mutated and repressed in T-ALL, and further experiments reveal that PTPN2 negatively regulates the JAK/STAT signaling (39). Other studies have found that *PTPN2* can act as an oncogene. *PTPN2* played tumor-promoting functions in B-cell lymphomas, and *Ptpn2* depletion decreased murine B-cell lymphoma cell proliferation and completely abolished the cancer *in vivo* (40). It was also shown that *PTPN2* expression levels are strongly associated with prognosis in patients with glioma and glioblastoma. Patients with a high expression of *PTPN2* tend to have a poor prognosis, suggesting that *PTPN2* can promote tumor development (41). Here, we show for the first time that PTPN2 plays a tumor-promoting function in KRAS-driven cancer.

It has been proven that KRAS is phosphorylated via Src, which alters the conformation of switch I and II regions, profoundly reducing the KRAS' binding affinity to RAF. In contrast, SHP2 dephosphorylates KRAS and thereby restores the affinity to RAF, which enhances the KRAS-mediated MAPK pathway. Inhibition of SHP2 promotes accumulation of phosphorylated KRAS and subsequently suppressing KRAS-RAF-MAPK signaling (32). Here, PTPN2 is identified as another phosphatase that dephosphorylates KRAS and regulates the activation of KRAS and its downstream signaling.

It has been suggested that the type of mutations in KRAS may have an influence on its ability to transform and the drug responses of cancer patients. SHP2 inhibitor RMC-4550, for example, has shown a potent inhibitory effect on the cancer cell lines bearing missense mutations in KRAS at Gly-12, but not Gly-13, and Gln-61 (42). In this study, we found it is interesting that the requirement of PTPN2 for KRAS activation is independent of the mutant *KRAS* isoforms, which included G12V (PaTu8988T), G13D (HCT-116), and Q61K (H460). Both PTPN2 and SHP2 can dephosphorylate KRAS, but their negative regulatory signal molecules are somewhat different. The underlying mechanism requires subsequent experiments to explore.

Recently, studies have shown that *Kras-*mutant-bearing cancers display features with reduced T helper 1 cells as well as reduced infiltration of cytotoxic cells via recruiting the myeloid-derived suppressor cells, which made *Kras-*mutant tumor resistant to immune checkpoint blockade (ICB) therapy (43). One mechanism is that *Kras*-driven cancers could down-regulate the interferon-γ (IFN-γ) and IFN-α responses, which are crucial in anti-PD-1 therapy in patients with cancer (43). It is interesting that loss of *Ptpn2* results in an increase in number and activation of CD8+ T cells, and enhancing of IFN-γ-mediated effects on antigen presentation (44, 45). Therefore *Ptpn2* has recently been identified as a novel cancer immunotherapy target in a CRISPR screening *in vivo*, where deletion of this gene increased the efficacy of immune checkpoint blockade therapy in melanoma (45). Moreover, one study has demonstrated that myeloid cell-specific loss of *Ptpn2* promotes inflammasome activation, resulting in protection from colorectal cancer (46). Thus, inhibition of PTPN2 could suppress KRAS cancer, whereas enhancing tumor immunity.

In summary, we identified PTPN2 is a key regulator of KRAS activation and signaling transduction. The results indicate that PTPN2 may be a novel therapeutic target for KRAS-driven cancers.

## Materials and methods

### Cell culture

Human lung (H460 and A549), pancreatic (PaTu8988T), colon (HCT-116 and DLD-1), fibrosarcoma (HT1080), and skin (SK-MEL-30) cancer cell lines and the HEK 293T cell line were obtained from the American Type Culture Collection (ATCC) and authenticated by ATCC and DSMZ using Short Tandem Repeat profiling analysis. These cells were detected periodically to ensure mycoplasma-free cells. HCT-116 and DLD-1 cells were cultured in RPMI-1640 medium (BasalMedia, Shanghai, China); HEK 293T, H460, PaTu8988T, and A549 cells were cultured in Dulbecco's modified Eagle's medium (BasalMedia, Shanghai, China), HT-1080 and SK-MEL-30 were cultured in minimal essential medium (BasalMedia, Shanghai, China). All media were supplemented with 10% fetal bovine serum (Gibco, Carlsbad, CA, USA), 1× penicillin/streptomycin, and the cells were cultured in a humidified 5% CO_2_ incubator at 37°C.

### DNA constructs

GFP-fused KRAS^G12D^, Myc-fused KRAS^G12D^, PTPN2 siRNA-1161–resistant (PTPN2-Res), and catalytically inactive mutation (PTPN2-D182A) plasmids were generated as previously described (6, 47). All constructs were confirmed by DNA sequencing before use.

### HEK 293T cell line stably expressing GFP-KRAS^G12D^

HEK 293T cells were maintained in Dulbecco's modified Eagle's medium supplemented with 10% fetal bovine serum at 37°C, 5% CO_2_. Transfection of GFP-KRAS^G12D^ was carried out using Lipofectamine 3000 reagent (Invitrogen) per the manufacturer's protocol. GFP-positive cells were sorted twice on a fluorescent-activated cell sorting (FACS) machine, and further placed on a 96-well flat-bottom tissue culture plate, one GFP positive cell/well. Cells were further cultured for 2 weeks, detached with trypsin-EDTA, and then cultured in a 6-well–plate. The GFP-KRAS^G12D^ expressing cell lines were used for further experiments.

### siRNA screening

siRNA screening was performed in GFP-KRAS^G12D^ HEK 293T cells using a pre-aliquoted Silencer siRNA library (Dharmacon; Human ON-TARGETplus, Lafayette, CO, USA) at 25 nm final concentration. siRNAs were arrayed into 96-well–plates (CellCarrier-96, PerkinElmer, Waltham, MA, USA) in duplicate. Gene silencing was induced for 72 h via reverse transfection using RNAiMAX reagent (Invitrogen, catalog number 13778). Cells with mislocalized GFP-KRAS^G12D^ were then screened using an Opera Phenix High-Content Screening System (PerkinElmer). Images were acquired for 17 fields with a ×40 water immersion objective using the GFP-confocal mode. The siRNA was listed in Table S1.

### Western blot analysis

To prepare whole-cell lysates, cells were washed twice with ice-cold phosphate-buffered saline (PBS) and lysed in SDS lysis buffer (100 mm Tris-HCl, 2% SDS, 10% glycerol, 50 mm dithiothreitol, 0.1% bromophenol blue, pH 6.8) supplemented with protease inhibitor cocktail and 2 mm phenylmethylsulfonyl fluoride. Proteins from the lysates were separated by SDS-polyacrylamide gel electrophoresis and Western blotted with the following antibodies: (*a*) anti-phospho–MEK1/2 (Ser-217/221) (catalog number 9121, 1:1000), anti-phospho–ERK1/2 (D13.14.4E; catalog number 4370, 1:1000), anti-GFP (catalog number 2555, 1:1000), anti-p44/42 MAPK (Erk1/2) (137F5) (catalog number4695S, 1:1000), anti-MEK1/2 (9122; 1:1000), and anti-Myc (71D10, catalog number 2278, 1:1000) (all from Cell Signaling Technology, Danvers, MA, USA); (*b*) anti-KRAS (catalog number WH0003845M1, 1:1000) and anti-PTPN2 (catalog number HPA046176, 1:1000) (both from Sigma-Aldrich), anti-[EP1845Y] sodium potassium ATPase (catalog number ab76020; 1:1000, from Abcam); (*c*) HRP-conjugated GAPDH (catalog number HRP-6004, 1:4000), HRP-conjugated β-actin (catalog number HRP-60008, 1:4000), and HRP-conjugated α-tubulin antibody (catalog number HRP-66031, 1:4000) (all from ProteinTech, Chicago, IL, USA). The secondary antibody was anti-mouse IgG, HRP-linked antibody (Cell Signaling Technology, 1:3000; catalog number 7076) or anti-rabbit IgG, HRP-linked antibody (Cell Signaling Technology, 1:3000; catalog number 7074). The Western blots images were developed with an Amersham Imager 600 (GE Healthcare, Boston, MA, USA). Each Western blot shown is a representative of a minimum of 2 independent experiments.

### Flow cytometry

The thymidine analog 5-bromo-2-deoxyuridine (BrdU) incorporation assay was performed according to the standard protocol of the manufacturer (BD Pharmingen™ BrdU Flow Kits; catalog number 559619). Cells were transfected with siRNA via reverse transfection using RNAiMAX reagent (Invitrogen, catalog number 13778) at a final concentration of 50 nm and seeded in 6-well–plates for 72 h. BrdU (10 μm) was added and incubated further for 3 h. Then, cells were washed three times with 1× PBS and fixed and permeabilized the cells with BD Cytofix/Cytoperm Buffer. Cell were further permeabilizated with Cytoperm Permeabilization Buffer Plus for 10 min on ice and re-fixed with Cytofix/Cytoperm Buffer for 10 min on ice. After incubation with DNase for 1 h at 37°C, cells were stained with FITC-conjugated anti-BrdU antibody for 30 min at room temperature. Before detecting by flow cytometry, cells were stained with nucleolus dye 7-amino-actinomycin D for 15 min. The percentage of BrdU-positive cells was counted and reckoned by using the GraphPad Prism 5.

Apoptosis was detected and quantified using the Annexin V Apoptosis Detection Kit APC (eBioscience, Waltham, MA, USA, catalog number 88800772). Briefly, after treatment with siRNA for 72 h, cells were harvested and washed with ice-cold PBS, and then suspended in Annexin binding buffer. Subsequently, cells were incubated with Annexin V-APC and propidium iodide for 15 min at room temperature in the dark and immediately analyzed using a BD LSRFortessa flow cytometer (BD Biosciences, Franklin Lakes, NJ, USA). Data were analyzed with the FlowJo (Tree Star) software.

### siRNA-mediated knockdown of KRAS and PTPN2

Cells were transfected with siRNA via reverse transfection using Dharmafect 1 Transfection Reagent (Dharmacon, catalog number T2001-01) or RNAiMAX reagent (Invitrogen, catalog number 13778) at a final concentration of 50 nm and seeded in 6-well–plates for Western blotting or apoptosis assays and 96-well–plates for cell viability assays. Transfected cells were collected 72 h after transfection for Western blot analysis as described above, and after 72 h for the CellTiter-Glo® Luminescent Cell Viability Assay (Promega) as described below. The siRNAs were obtained from GenePharma Corporation (Shanghai, China) and the sequences used for KRAS siRNA and PTPN2 siRNA experiments are as follows: negative control, sense UUCUCCGAACGUGUCACGUTT and antisense ACGUGACACGUUCGGAGAATT; KRAS siRNA-244, sense GCCUUGACGAUACAGCUAATT and antisense UUAGCUGUAUCGUCAAGGCTT; KRAS siRNA-585, sense GGACUUAGCAAGAAGUUAUTT and antisense AUAACUUCUUGCUAAGUCCTT; KRAS siRNA-643, sense GGUGUUGAUGAUGCCUUCUTT and antisense AGAAGGCAUCAUCAACACCTT; PTPN2 siRNA-432, Sense CAAAGGAGUUACAUCUUAATT and antisense UUAAGAUGUAACUCCUUUGTT; and PTPN2 siRNA-1161, sense GCCUUUGAUCAUUCACCAATT and antisense UUGGUGAAUGAUCAAAGGCTT.

### Cell viability assay

Cell viability assays were carried out using the CellTiter-Glo® Luminescent Cell Viability Assay as previously described (48). H460, PaTu8988T, HCT-116, A549, DLD-1, HT-1080, and SK-MEL-30 cells were transfected with siRNA via reverse transfection using Dharmafect 1 or RNAiMAX at a final concentration of 50 nm and seeded in regular 96-well–plates at a density of 5 × 10^3^ to 1 × 10^4^ cells/well. After 72 h incubation, cell viability was measured using the CellTiter-Glo reagent. The luminescence was detected using an Envision plate reader (PerkinElmer).

### KRAS activity assay

KRAS activity was determined using RAS activation assay kit (EMD Millipore, 17–218, Burlington, MA, USA) according to the manufacturer's protocol with minor modifications. Briefly, cells were lysed in Pierce IP Lysis Buffer (Thermo Scientific, catalog number 87788) supplemented with protease and phosphatase inhibitors (Roche Applied Science), and lysates were further incubated with 5 μg of RAF-1 RBD-agarose beads for 8 h at 4 °C. After washing the agarose beads three times with Pierce IP Lysis Buffer, the activated KRAS (GTP-RAS) bound to RAF-1 RBD-agarose beads was released by the addition of SDS lysis buffer. Finally, samples were subjected to Western blotting analysis as previously described and blots were probed using an anti-KRAS antibody (Sigma, 1:1000).

### Immunoprecipitation and immunoblotting

HEK 293T cells were transfected with siRNA via reverse transfection using Dharmafect 1 at a final concentration of 50 nm and seeded in regular 6-well–plates at a density of 1 × 10^6^ cells/well. After 24 h incubation, cells were transiently transfected the Myc-tagged KRAS^G12D^ plasmid using the Lipo6000 Transfection Reagent (Beyotime, Shanghai, China, catalog number C0526) per the manufacturer's protocol and further cultured for 48 h. Cells were lysed in Pierce IP Lysis Buffer supplemented with protease and phosphatase inhibitors (Roche), and lysates were further incubated with 10 μg of anti-Myc tag antibody (agarose) (Abcam, Cambridge, England, UK, catalog number ab1253) or IgG-agarose beads (Abcam, catalog number ab104155) for 8 h at 4 °C. After washing the agarose beads three times with Pierce IP Lysis Buffer, the Myc-tagged-KRAS bound to agarose beads was released by the addition of SDS lysis buffer. Finally, samples were subjected to Western blotting analysis as previously described and blots were probed using an anti-phosphotyrosine antibody (Cell Signaling Technology, catalog number 9411, 1:1000) or anti-Myc antibody (Cell Signaling Technology, 1:1000).

### KRAS protein stability

GFP-KRAS^G12D^ HEK 293T cells and H460 were transfected with siRNA via reverse transfection using RNAiMAX at a final concentration of 50 nm and seeded in regular 12-well–plates at a density of 2.5 × 10^5^ cells/well. Forty-eight h later fresh medium containing cycloheximide (50 μg/ml) was added. At the indicated time intervals, cells were washed twice with ice-cold PBS and lysed in SDS lysis buffer supplemented with protease inhibitor cocktail and 2 mm PMSF. Finally, samples were subjected to Western blotting analysis as previously described.

### Analyses of the association of the KRAS mutation, KRAS expression, and PTPN2 expression in PAAD, LUAD, or COAD clinical outcome

The KRAS mutation, PTPN2 expression, and clinical information of KRAS-related cancers were extracted from the TCGA database. This dataset contains survival data with clinical information, KRAS mutations, KRAS mRNA expression counts, and PTPN2 mRNA expression counts. The groups were separated by the mean expression level of the group. Overall survival stratified by expression levels of the gene of interest was evaluated using Kaplan–Meier analysis, and comparisons between groups were evaluated using log-rank tests. *p* < 0.05 was considered statistically significant.

## Statistical analysis

Data of continuous variables are presented as mean ± SD. Comparisons between treatments were analyzed by one-way ANOVA followed by Tukey's test using GraphPad Prism 5. *p* < 0.05 was deemed statistically significant.

## Data availability

All data relevant to this study are included within this manuscript.
